# Differential expression of ghrelin and GHSR via the mTOR pathway during the dynamic carcinogenic process involving oral, potentially malignant disorders

**DOI:** 10.1042/BSR20192102

**Published:** 2019-12-17

**Authors:** Jianing Lou, Lin Liu, Weizhen Zhang, Zengtong Zhou, Yuan Fan

**Affiliations:** 1 Department of Stomatology, Shanghai General Hospital of Nanjing Medical University, Shanghai 201620, China; 2 Jiangsu Key Laboratory of Oral Diseases, Nanjing Medical University, Nanjing, China; 3 Department of Surgery, Medical School, University of Michigan, Ann Arbor, MI 48109, U.S.A.; 4 Department of Oral Medicine, Shanghai Key Laboratory of Stomatology, Shanghai Ninth People’s Hospital, Shanghai Jiao Tong University School of Medicine, Shanghai 200011, P.R. China; 5 Department of Oral Medicine, Affiliated Hospital of Stomatology, Nanjing Medical University, Nanjing, China

**Keywords:** carcinogenesis, Ghrelin receptor, ghrelin, mammalian target of rapamycin, oral potentially malignant disorders

## Abstract

The purpose was to explore the sequence changes in ghrelin and GHSR in the mTOR signaling pathway during carcinogenesis involving oral, potentially malignant disorders (OPMD). The samples were confirmed through *in vivo* pathologic tissue screening and diagnosis. The immunohistochemical method was used to detect the expression of the ghrelin/growth hormone secretagogue receptor (GHSR) protein. The expression of ghrelin, GHSR 1α, GHSR 1β, and mammalian target of rapamycin (mTOR) RNA were detected by real-time PCR. The expression of ghrelin, GHSR, mTOR, and phosphorylated mTOR (phosphor-mTOR) protein were detected by Western blot. The expression of ghrelin/GHSR increased gradually in the dynamic process of OPMD carcinogenesis. There was a correlation between the increase in ghrelin, GHSR, mTOR, and phospho-mTOR. The *in vivo* expression of ghrelin/GHSR protein was the most apparent pathologic change from normal-to-mild, moderate, and severe dysplasia, and finally to the dynamic process from normal-to-mild-to-moderate dysplasia. The *in vitro* cell experiments based on QPCR results also proved that GHSR 1a functional receptor of ghrelin had a peak expression in LEUK-1 cells. In conclusioin, the close relationship between ghrelin and OPMD carcinogenesis can be used as a new biological target to assess the carcinogenesis of OPMD.

## Introduction

Oral cancer accounts for 3.1–4.6% of malignant tumors in China, and the incidence is increasing. Approximately 90% of oral malignancies are squamous cell carcinomas, which mostly undergo a dynamic development process from normal cells to oral, potentially malignant disorders, and then to cancer. The 5-year survival rate of oral cancer is approximately 50%, while the rate of early oral cancer is >80% [1,2]. The World Health Organization (WHO) refers to diseases that may cause oral cancer as oral potentially malignant disorders (OPMDs) [3,4]. The pathogenesis and carcinogenesis of most OPMDs are not clear. There is generally no specific treatment, and there is a lack of effective chemoprophylaxis for cancer [5,6]. The poor prognosis and multiple and recurrent conditions of OPMDs may result in significant physical discomfort and mental distress among patients. Thus, an early diagnosis of OPMDs with a high risk of carcinogenesis is of great significance in preventing the occurrence of oral malignant tumors [7,8].

Many biomarkers were used for differentiation between OPMD and malignant tumors. Saliva protein biomarkers were used to detect oral squamous cell carcinoma in a high-risk population in Taiwan [9]. Podoplanin as a type I transmembrane sialomucin-like glycoprotein could be different among oral leukoplakia, oral submucous fibrosis and oral squamous cell carcinoma with that in normal buccal mucosa [10]. Higher levels of serum squamous cell carcinoma antigen may serve as a marker for dysplasia and progression to oral carcinogenesis [11]. In 1999, Kojima [12] discovered and successfully isolated a small peptide from the stomachs of experimental rats that can activate the growth hormone secretagogue receptor (GHSR), and named the peptide ghrelin. Ghrelin promotes the release of growth hormone (GH), stimulates food intake, regulates energy metabolism, and plays a role in hormone secretion, glucose and lipid metabolism, immune regulation, cell proliferation, and apoptosis [13]. Mammalian target of rapamycin (mTOR), as a classical intracellular signal transduction molecule, regulates cell cycle processes and cell growth by sensing energy homeostasis changes [14]. Indeed, mTOR has become a commonly used tool for studying the regulation of cell proliferation due to its anti-proliferation characteristics. The significant role of this pathway in tumor development deserves our attention.

The purpose of the present study was to explore the possibility of ghrelin and ghrelin receptors as potential targets for oral mucosal malignant transformation, and the correlation between ghrelin and the mTOR signaling pathway in the regulation of OPMDs.

## Materials and methods

### Tissue sampling

The pathologic tissues used in the present study were obtained from the Department of Oral Pathology of the Ninth People’s Hospital affiliated to the School of Medicine at Shanghai Jiaotong University. Normal oral mucosal tissue, mild, moderate, and severe oral mucosal epithelial dysplasia, and oral squamous cell carcinoma (20 cases each) were included. After the samples were sectioned in triplicate, one section was stained with hematoxylin–eosin (HE) for confirmation of the pathologic diagnosis, and the other two sections were numbered and saved for immunohistochemistry.

The cell lines used in the present study were HOK, LEUK1, and CAL27. The samples were obtained from the Professional Technical Service Platform of Shanghai Oral and Maxillofacial Tumor Tissue Samples and Biological Information Database (18DZ2291500). The cultured cell lines were transferred to two culture flasks, one for real-time PCR and the other for Western blot.

### Hematoxylin–eosin

Hematoxylin–eosin was performed according to previous study [15]. Hematoxylin solution was dyed for 5–20 min, rinsed under running water, differentiated by differentiation solution for 30 s, soaked with tap water for 15 min or warm water for 5 min, stained with eosin for 2 min, rinsed under running water, soaked with tap water for 5 min, and then washed in an alcohol gradient for dehydration (95%, 100%I, and 100%II for 1 min each). Xylene I and II were added for 10 min, respectively. The tissue section was sealed with neutral gum, dried in a 60°C oven, and observed under the microscope.

### Immunohistochemistry

Immunohistochemistry was performed with two primary antibodies (rabbit anti-ghrelin, TDY532C; Tdybio, city, Germany and rabbit anti-GHSR, 13309-1-AP; Proteintech, city, Germany). Briefly, the procedure was as follows [16]: (1) de-waxing in xylene and dehydration with alcohol; (2) incubating in citrate buffer (0.01 mol/l, pH 6.0), microwaving (power level 3) for 10–15 min and washing with PBS (0.01 M, pH 7.4; (3) blocking the activity of endogenous peroxidase and washing in PBS; (4) sealing with 5% BSA/0.01 M PBS, then adding the diluted antibody solution and incubating at 4°C overnight; (5) reheating the wet box at room temperature for 15 min the next day and washing with PBS; (6) adding the second antibody and incubating at room temperature for 30 min and washing with PBS; (7) applying the DAB solution for 3–5 min, until the yellow–brown spots were observed under the microscope; (8) counterstaining with hematoxylin (Sigma, St. Louis, MO, U.S.A.) for 3 min, then washing with distilled water for 30 s; (9) using hydrochloric acid and alcohol to differentiate for 5 s, then washing with distilled water for 15 min; and (10) dehydrating, sealing, baking, and observing under a microscope.

### Cell line cultures

The human cell lines (HOK, LEUK, and CAL27) were used. The cells were cultivated in 10% fetal bovine serum and 1% antibiotics in a 37°C water bath under 5% CO_2_. When the cells covered 70–80% of the growth surface, some cells were reserved for cryopreservation. In the logarithmic growth phase, 1 ml of 0.25% trypsin was added to digest the cells and 10% fetal bovine serum was added to terminate digestion when the cells were contracting and becoming round. The cell suspension was centrifuged at room temperature at l000 rpm [*g* values preferred] for 5 min, then transferred to two culture flasks containing 10% fetal bovine serum for further culture at 37°C and 5% CO_2_.

### Real-time PCR

A Takara reagent kit (Takara, Tokyo, Japan) was used to extract the total RNA [17]. The RNA was reverse-transcribed into cDNA by using a PrimeScript RT reagent kit (Takara). The expression of ghrelin, GHSR1α, GHSR1β, and mTOR RNA was detected by real-time PCR using a quantitative PCR system (ABI 7300, company, city, state, U.S.A.) with SYBR Premix. After the reaction, real-time PCR amplification and thawing curves were confirmed, and the 2^−ΔΔ*C*t^ values were calculated.

### Western blot

The expression of ghrelin, GHSR, mTOR, and phospho-mTOR protein was detected by Western blot. The cells were homogenized by mixing with radioimmunoprecipitation assay (RIPA; Beyotime, Shanghai, China) containing protease inhibitors (100:1). The total cellular protein concentration was determined using the Pierce™ BCA Protein Assay Kit (city, state, country), and nuclear protein was quantified according to the Bio-Rad Protein Assay (city, state, country). Proteins were resolved by sodium dodecyl sulfate-polyacrylamide gel electrophoresis (SDS-PAGE) with 10% polyacrylamide gels, then transferred to polyvinylidene difluoride (PVDF) membranes (Millipore, Billerica, MA, U.S.A.). Next, the PVDF membranes were blocked in 5% bovine serum albumin (BSA) for 2 h at room temperature. The blots were then incubated in primary antibody (1:1000, ab154845; Abcam, city, state, U.S.A.) overnight at 4°C, washed with TBST, and incubated with secondary antibodies (1:1000; Zhongshan Golden Bridge, Beijing, China) for 1 h at room temperature. The blots were then washed again. Finally, the protein bands were detected by using Immobilon Western Chemiluminescent HRP substrate (Millipore) and visualized using the Image Quant LAS 4000 mini-imaging system (GE, city, New York, U.S.A.). The average gray value was measured by ImageJ for Windows software, and the internal reference ratio of each target protein was calculated.

### Statistical analysis

All experimental data were statistically analyzed using IBM SPSS Statistics software (version 25.0, city, state, U.S.A.). The measurement data are expressed as the means ± standard deviations (SD). The differences between the multiple groups were evaluated using ANOVA. There was a significant difference between the two groups (*P* < 0.05).

## Results

### Specimen screening and pathologic review in the process of human oral mucosa carcinogenesis

**Figure 1:**
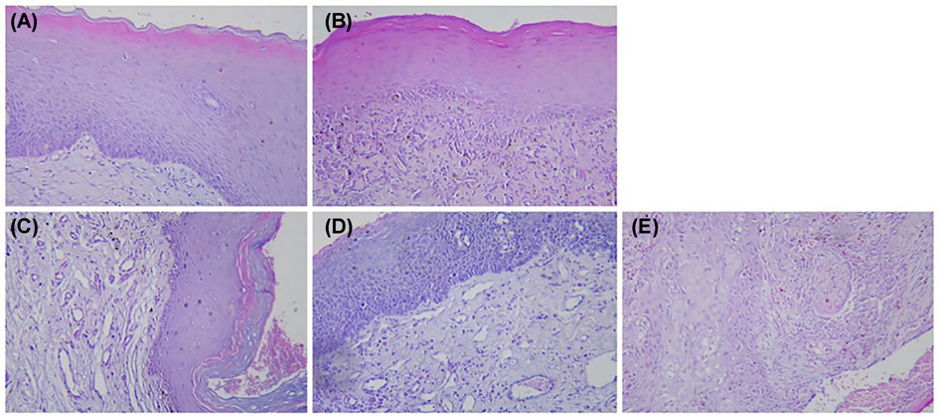
HE staining contrast HE staining of normal oral mucosa (**A**), mild dysplasia (**B**), moderate dysplasia (**C**), severe dysplasia (**D**), and epithelial cell carcinoma (**E**) (×200).

HE staining showed that with aggravation of epithelial dysplasia (i.e., the sequence changes from normal-to-mild, -moderate, and -severe dysplasia, then to oral cancer, the polarity of basal epithelial cells gradually disappeared in the oral mucosal epithelial cortex, and more than one layer of basal cells appeared. The epithelial nail process was dripping, the epithelial nucleus was deeply stained, and the nucleolus was clearly enlarged. The mitotic phase increased and the ratio of the nucleus-to-cytoplasm increased. The cell level was disordered, the number of abnormal cells increased, and the adhesion of cells decreased. Mitotic phases also appeared in the superficial one-half of the epithelium. Single or clustered keratinization occurred in spinous layers (Figure 1).

### Differential expression of ghrelin and GHSR in human oral mucosa during carcinogenesis

**Figure 2:**
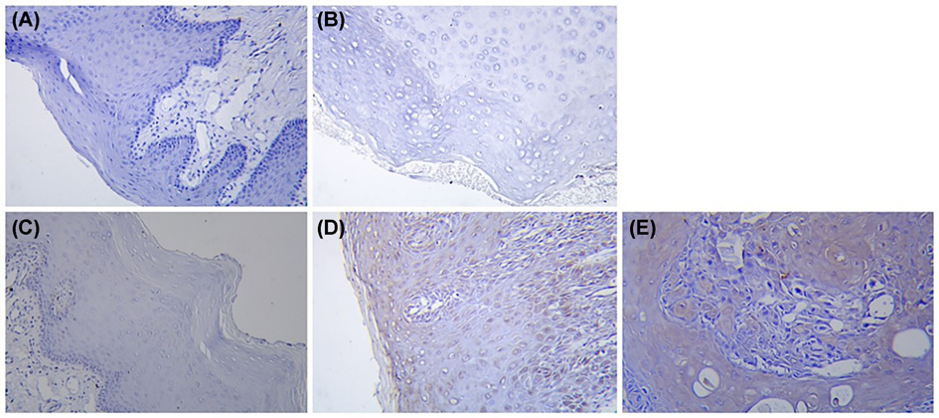
Ghrelin IHC contrast Ghrelin IHC of normal oral mucosa (**A**), mild dysplasia (**B**), moderate dysplasia (**C**), severe dysplasia (**D**), and epithelial cell carcinoma (**E**) (×200).

**Figure 3:**
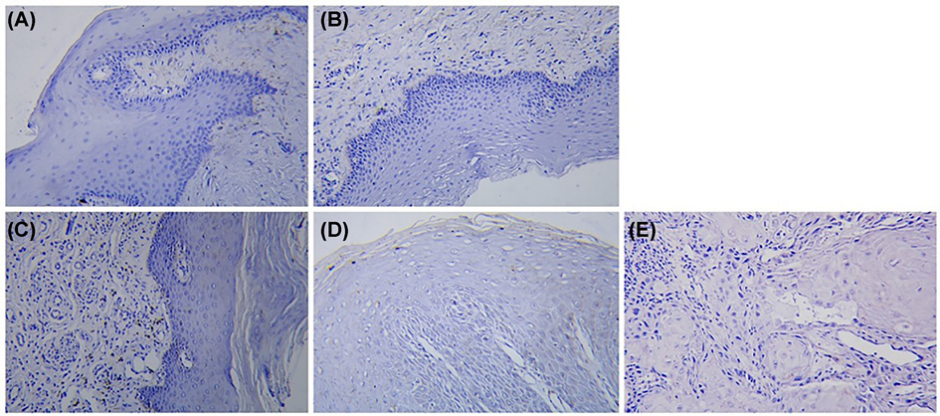
GHSR IHC contrast GHSR IHC of normal oral mucosa (**A**), mild dysplasia (**B**), moderate dysplasia (**C**), severe dysplasia (**D**), and epithelial cell carcinoma (**E**) (×200).

Immunohistochemical staining under the light microscope showed that specific positive staining of the target protein, ghrelin, and the receptor, GHSR, could be detected (Figures 2 and 3). With the aggravation of the degree of epithelial dysplasia, that is, the sequence changes from normal to light, medium and severe dysplasia and oral cancer, the expression of GHSR gradually increased and was positively correlated with Ghrelin, and the positive staining area gradually expanded.

**Figure 4:**
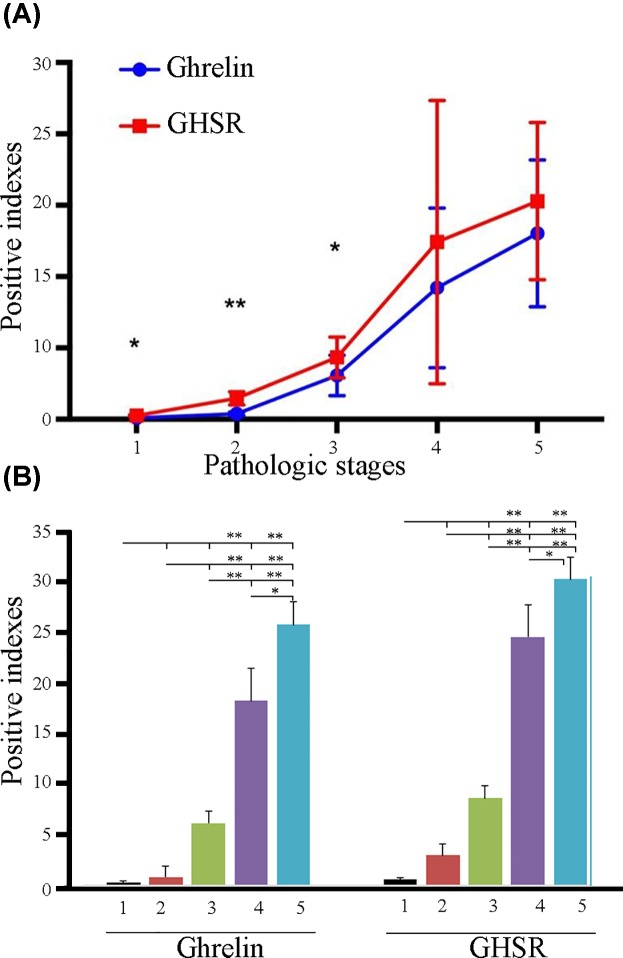
Comprehensive analysis of ghrelin and GHSR Comprehensive analysis of positive index between ghrelin and GHSR group (*t* test) (**A**); comparison of positive indices of ghrelin and GHSR in different pathologic stages (one-way ANOVA) (**B**). In the Figures, 1–5 indicated normal, mild dysplasia, moderate dysplasia, severe dysplasia, and OSCC, respectively. * indicated *P* < 0.05, ** indicated *P* < 0.01

According to the grayscale statistical analysis of scanning image software, the positive indices of ghrelin and GHSR gradually increased from normal epithelium-to-mild, -moderate, and -severe dysplasia, then culminating in oral cancer (Figure 4A). The positive ghrelin index gradually increased from 0.174 in normal epithelium to 0.765766667 in mild dysplasia, 6.156566667 in moderate dysplasia, 18.41913333 in severe dysplasia, and 26.05422 in oral cancer. The GHSR positive index gradually increased from 0.524166667 in normal epithelium to 2.947433333 in mild dysplasia, 8.678533333 in moderate dysplasia, 24.84486667 in severe dysplasia, and 30.58046667 in oral cancer. The most obvious changes of expression in Ghrelin and GHSR protein were in the pathological changes of moderate to severe dysplasia.

The *t*-test results of the positive index between ghrelin and GHSR in different pathologic stages showed that GHSR was higher than ghrelin in different pathologic stages. There were significant differences between the two groups with normal (*P* = 0.04) and moderate dysplasia (*P* = 0.025), extremely significant differences with mild dysplasia (*P* = 0.00), and no significant differences with severe dysplasia (*P* = 0.38) and oral cancer (*P* = 0.342) (Figure 4A).

The ANOVA results showed that ghrelin and GHSR positive indices in severe dysplasia and cancer with normal, mild and moderate dysplasia were significantly different (*P* < 0.01), and the ghrelin positive index in the severe dysplasia and cancer data were also significantly different (*P* < 0.05; Figure 4B).

### Quantitative detection of mTOR, ghrelin, and receptor RNA in human oral mucosal cell lines during carcinogenesis

**Figure 5:**
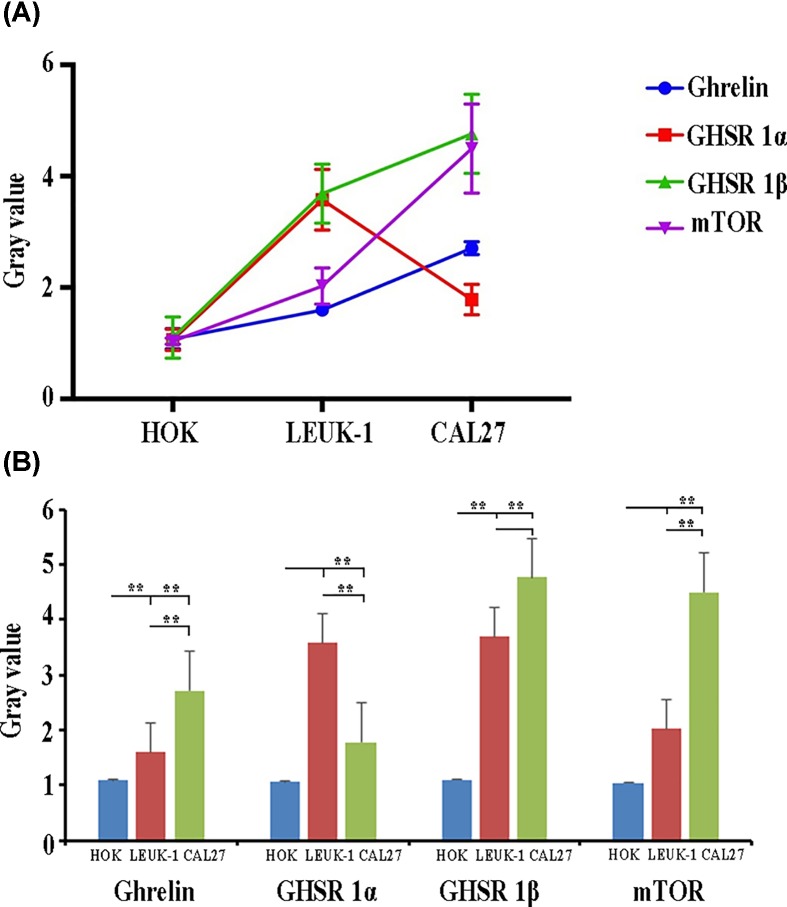
Analysis of ghrelin, GHS-R1α, GHS-R1β, and mTOR RNA in HOK, LEUK1, and CAL27 cells Dynamic expression trend analysis of ghrelin, GHS-R1α, GHS-R1β, and mTOR RNA in HOK, LEUK1, and CAL27 cells (**A**); Analysis of the differential expression of ghrelin, GHS-R1α, GHS-R1β, and mTOR RNA in HOK, LEUK1, and CAL27 cells (**B**). ** indicated *P* < 0.01.

The levels of ghrelin, GHSR1 β, and mTOR RNA showed an increasing trend in the HOK, LEUK1, and CAL27 cell lines, respectively; however, the level of GHSR1α expression in the CAL27 cell line showed a peak value, which was lower than the LEUK1 cell line, but higher than the LEUK1 cell line (Figure 5A).

As compared with the HOK cell line, ghrelin, GHSR1α, and GHSR1β showed significantly higher levels in the LEUK1 cell line (*P* = 0.004, *P* = 0.00, *P* = 0.001), while mTOR showed no significant increase (*P* = 0.052). Compared with the HOK cell line, ghrelin, GHSR1β, and mTOR were significantly increased in the CAL27 cell line (*P* = 0.00, *P* = 0.00, *P* = 0.00) and GHSR1α showed no significance (*P* = 0.053). Compared with LEUK1 cells, ghrelin and mTOR were significantly increased in the CAL27 cell line (*P* = 0.00, *P* = 0.001), the rise in GHSR1β was not significant (*P* = 0.056), and GHSR1α was significantly lower (*P* = 0.001) (Figure 5B).

### Semi-quantitative detection of mTOR, ghrelin, and receptor proteins in human oral mucosal cell lines during carcinogenesis

**Figure 6:**
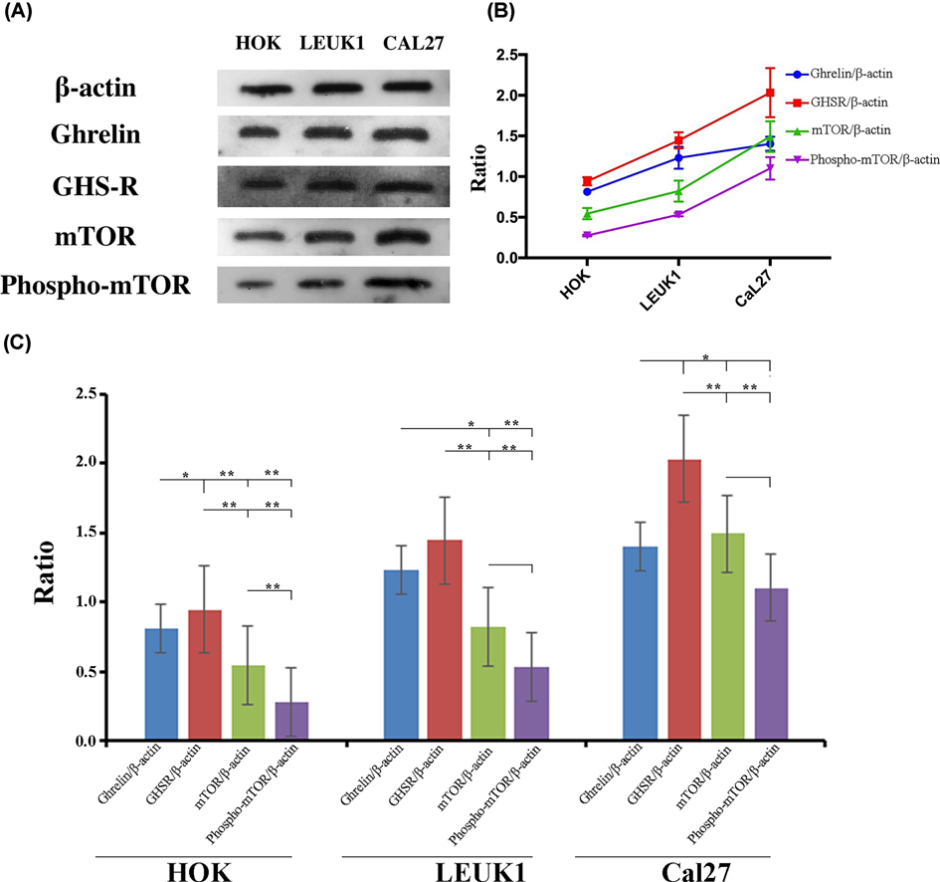
Analysis of ghrelin, GHS-R, mTOR, and phospho-mTOR proteins in HOK, LEUK1, and CAL27 cells Gray value of ghrelin, GHS-R, mTOR, and phospho-mTOR in HOK, LEUK1, and CAL27 cell lines on Western blot (**A**). Dynamic expression trend analysis of ghrelin, GHS-R, mTOR, and phospho-mTOR proteins in HOK, LEUK1, and CAL27 cells (**B**). Analysis of the differential expression of ghrelin, GHS-R, mTOR, and phospho-mTOR proteins in HOK, LEUK1, and CAL27 cells (**C**). * indicated *P* < 0.05, ** indicated *P* < 0.01

Specific positive bands of related target proteins were detected. The test results of β-actin were normal positive. Western blot analysis showed that the expression of ghrelin, GHSR, mTOR, and phospho-mTOR in the malignant cell lines with HOK, LEUK1, and CAL27 sequences increased gradually and showed a consistent trend (Figure 6A,B).

Ghrelin, GHSR, mTOR and phospho-mTOR levels of the CAL27 cell line were significantly higher than the HOK cell line (*P* = 0.008, *P* = 0.01, *P* = 0.006, *P* = 0.002). As compared with HOK, LEUK1 cells showed significantly higher levels of ghrelin and phosphorylation of mTOR (*P* = 0.021, *P* = 0.05), but no significant increases in GHSR and mTOR (*P* = 0.073, *P* = 0.138). Compared with the LEUK1 cell line, the CAL27 cell line showed no significant increase in ghrelin and GHSR (*P* = 0.159, *P* = 0.051), while both mTOR and phosphorylated mTOR showed significant increases (*P* = 0.017, *P* = 0.006) (Figure 6C).

## Discussion

GHSR is a G protein-coupled receptor and there are currently two known subtypes (GHSR 1a and GHSR 1β) [18]. GHSR1a consists of 366 amino acids and has 7 complete transmembrane segments, which are the main functional receptors. The GHSR1b subtype has 289 bases, including only the first 5 transmembrane segments [19]. Ghrelin is an endogenous ligand of GHSR 1α [20]. Through selective splicing and post-translational modification, the ghrelin gene generates a variety of peptide hormones, including acyl-ghrelin, des-acyl-ghrelin, obestatin, and c-ghrelin [21]. Only acyl-ghrelin can bind and activate its receptor, GHSR 1α [22]. The functions of these peptide hormones involve almost all major systems of the body, including glucose and lipid metabolism, energy balance, gastrointestinal function, immunity and inflammation, memory, anxiety, depression, cardiovascular function, embryonic development, and cell proliferation, differentiation, and apoptosis, in addition to the release of growth hormone discovered in the early stage [23,24]. The diversity of the ghrelin gene products and functions indicates its potential application in the prevention and treatment of various human diseases [25].

Although the degree of epithelial dysplasia is currently considered the gold standard for judging the risk of cancer, the subjectivity of diagnostic judgment and the inconsistency between diagnostic judgment and the degree of cancer risk also exist according to this index [26,27]. Therefore, whether or not epithelial dysplasia is a significant risk factor influencing the prognosis of cancer is still controversial [28]. To objectively and characteristically identify patients at high risk for cancer, the search for objective cancer risk markers can help us adopt corresponding strategies to reduce the incidence and mortality of oral cancer [29]. Previous studies involving oral cancer markers were mainly animal experimental studies and clinicopathologic studies on head and neck squamous cell carcinoma [30–32]; however, few clinical cohort studies with a focus on molecular epidemiology and based on a large sample size and long-term follow-up have been conducted involving biomarkers for oral, potentially malignant lesions [33,34].

Our study findings were confirmed by immunohistochemistry, Western blot, real-time PCR, and other studies on the differential expression of ghrelin and receptor proteins in the process of oral mucosal cancer *in vivo* and the quantitative detection of mTOR, ghrelin and receptor RNA and protein quantification *in vitro* during the process of oral mucosal cancer. Ghrelin promotes oral tumor cell proliferation by modifying GLUT1 expression [35]. Ghrelin is a potential molecular marker of adrenal carcinogenesis *in vivo* and *in vitro* [36]. Ghrelin and its receptor, GHSR, were also gradually increased in the dynamic process of OPMD carcinogenesis. The results suggested that ghrelin and GHSR play a vital role in the process of OPMD carcinogenesis and may become a new biological target for the determination of OPMD carcinogenesis; however, the causal relationship between ghrelin/GHSR and OPMD carcinogenesis is still unclear.

In normal keratinocytes (HOK), oral abnormally proliferating cells (LEUK1), oral cancer cells (CAL27), and sequence malignant cell lines, ghrelin, GHSR, mTOR, and phospho-mTOR showed the same increasing trend and correlation. A previous study found that activation of the mTOR-Akt signaling pathway induced by Rictor contributes centrally to oral carcinogenesis [37]. AMPK and Akt/mTOR signaling showed a key role in cisplatin-resistant human oral cancer CAR cells [38]. Our results suggested that ghrelin/GHSR may mediate the mTOR/phospho-mTOR signaling pathway to regulate OPMD carcinogenesis. Mild and moderate dysplasia in patients with OPMD may be the most important stage at which ghrelin/GHSR expresses a regulation and biological effect, and ghrelin/GHSR and mTOR may play a critical role in the carcinogenesis of OPMD.

IHC *in vivo* experiments confirmed that ghrelin and GHSR positive indices in severe dysplasia and cancer stage had very significant differences with normal cells, and mild and moderate dysplasia, respectively. The ghrelin positive index in severe dysplasia and cancer also had a significant difference. It suggested that the mild-to-moderate dysplasia stage of OPMD may be a critical period in which ghrelin/GHSR plays a regulatory role and produces biological effects. QPCR cell *in vitro* experiments also prove the function of ghrelin GHSR 1α-related receptors on cells expressing LEUK-1 peak, showing that cells and biochemical changes of abnormal epithelial hyperplasia may be the biological effect of the ghrelin/GHSR important stage.

Ghrelin, GHSR, and mTOR-related molecules were closely related to the dynamic changes of oral carcinogenesis, suggesting that the ghrelin/mTOR pathway could become a potentially valuable molecular pathway to monitor the malignant transformation of OPMD. The related indicators were expected to be applied in molecular epidemiology to carry out non-invasive screening of OPMD malignant transformation in healthy people, which was worthy of a new round of exploration on the specific upstream and downstream molecular mechanisms. As a new potential biomarker, ghrelin can predict the malignant trend of OPMD, including OLP, and can be used as a monitoring index for non-invasive screening of malignant changes of OPMD in the healthy population by molecular epidemiology. An intensive study is warranted involving the mechanism underlying the cell signaling pathway in OPMD.

## Availability of data and material

The datasets used and/or analyzed during the current study are available from the corresponding author on reasonable request.

## Abbreviations

GH: growth hormone
GHSR: growth hormone secretagogue receptor
mTOR: mammalian target of rapamycin
OPMD: oral, potentially malignant disorders
PVDF: polyvinylidene difluoride
